# Kentucky State Medical Society

**Published:** 1852-12

**Authors:** 


### EDITORIAL DEPARTMENT.

*Kentucky State Medical Society.* — A State Medical Society in Kentucky was organized in 1851, and the first regular annual meeting was held at Louisville, on the 20th of October, 1852.

This meeting was attended by a large delegation from different parts of the state, and continued in session three days. Great interest and entire harmony characterized the meeting. On the evening of the last day of the session, a sumptuous supper was served at the Louisville Hotel, in behalf of the Physicians of Louisville, at which the members of the Society residing out of the city, together with several distinguished citizens of Louisville, not members of the medical profession, were present as invited guests. Speeches were made, and the festivities of the evening were conducted with a degree of good taste in keeping with the elegance of the entertainment.

Several valuable papers were contributed during the session of the society. Prof. Miller, of the University of Louisville, read a highly interesting paper, as chairman of the committee on obstetrics. The subjects taken up by Prof. M. were chloroform in midwifery, and the use of the speculum in uterine diseases. These subjects were treated in a manner worthy of one of the most able and accomplished obstetrical teachers and practitioners living. The sentiments of Dr. Lee, of London, with respect to the speculum, were fully considered, and satisfactorily disposed of.

A report on the history of surgery in Kentucky, was read by Prof. Gross, of the University of Louisville. With respect to this most interesting and able paper, we adopt the language of Dr. Bell, co-editor of the Western Jour. of Med. and Surg.: "This admirable report, one of the finest contributions that has been made to the medical literature of Kentucky, excited the deepest interest; and although expectations were very high, we believe the Professor transcended all anticipations. The report contains the most complete history of Kentucky surgery that has been written, and it would be considered a first class paper among the *Eloges* of the French institute."

The report embraces a pretty full biography of Dr. McDowal, to whom belongs the honor of having first performed the operation of ovariotomy. Dr. McDowal was a native and resident of Kentucky, and the general impression, if we mistake not, is, that he became the originator of ovariotomy in consequence rather of his recklessness, than as the result of a careful pre-consideration of the reasons which should justify undertaking an operation of so much magnitude. This, at all events, we must confess to have been our own impression on the subject. It appears, however, from the biography by Prof. Gross, that an idea of that kind does great injustice to the character of the distinguished surgeon, whose name will ever hold a conspicuous place in the medical history of our country. Dr. McDowal was educated at Edinburgh, where he listened to the lectures of the talented and eloquent John Bell. While pursuing his studies under the guidance of that bold surgeon, he determined, whenever a favorable opportunity should offer in practice, to undertake the removal of ovarian disease by the knife. He put this design into execution in the first case that presented itself after returning home. He operated subsequently several times with remarkable success. The late President Polk, when about fourteen years of age, was operated on for stone, by Dr. McDowal. Prof. Gross, in connection with his report, exhibited the stone to the members of the society, and read a letter from Mr. Polk, while a member of congress, expressing his grateful sentiments in language as honorable to the character of the writer, as it must have been gratifying to the distinguished surgeon to whom it was addressed.

Religious fervor was a prominent trait in the character of Dr. McDowal. He trusted much in the efficacy of prayer for the safety of the patients who intrusted their lives to his skill; and his piety led him to attribute his remarkable success as a surgeon to the special blessings of Providence.

The papers contributed by Professors Gross and Miller will be published in the Transactions of the society, and will form most acceptable contributions to American medical literature.

Dr. S. S. Bell, one of the editors of the Western Journal of Medicine and Surgery, as chairman of committee on *Case-Book*, made a report urging upon the members of the society the importance of keeping records of cases falling under their observation. Dr. B. cited the example of the late Sir W. F. Chambers, of London, who kept a minute record of his cases in public and private practice, and at the time of his decease, had filled *sixty-seven quarto volumes*, each of 400 pages. These records were all written in latin.

A resolution was adopted providing for the publication of a case-book for the use of the members of the society.

Dr. Bell remarks: "It will be a proud day for medical science and practice in Kentucky, when all the practitioners of the state commence the use of the case-book. Those who begin it will find such benefits to themselves and patients, that the practice will not be abandoned speedily."

Of the benefits of keeping records of cases too much cannot be said. If materials thus collected are never made use of, the advantages will vastly overbalance the labor. It is in this way that habits of accurate observation are to be cultivated with the greatest success; and it is in this way alone that clinical experience can be rendered reliable and truly valuable.

Dr. Chipley, of Lexington, was elected president of the Kentucky State Medical Society, and a vice-president elected for the city of Louisville, and for each county in the state. Dr. S. S. Bell, the talented co-editor of the Western Journal of Med., was chosen vice-president from Louisville.

