# Errata

**Published:** 1853-01

**Authors:** 


*Errata.* — December No. page 452, 20th line, and several times in that article, for *ecrema* read *eczema.* Page 453, 15th line, for *grains*, read *grammes.* Page 453, 15th line, for *conscientiously* read *continuously.*

